# Structural remodeling of AAA+ ATPase p97 by adaptor protein ASPL facilitates posttranslational methylation by METTL21D

**DOI:** 10.1073/pnas.2208941120

**Published:** 2023-01-19

**Authors:** Saša Petrović, Yvette Roske, Biria Rami, Mai Hoang Quynh Phan, Daniela Panáková, Udo Heinemann

**Affiliations:** ^a^Max Delbrück Center for Molecular Medicine 13125, Berlin, Germany; ^b^Institute for Chemistry and Biochemistry, Freie Universität Berlin 14195, Berlin, Germany

**Keywords:** AAA+ ATPase, p97, protein methylation, protein remodeling

## Abstract

p97 is an essential AAA+ ATPase that extracts and unfolds substrate proteins from membranes and protein complexes. Through its mode of action, p97 contributes to various cellular processes, such as membrane fusion, ER-associated protein degradation, DNA repair, and many others. Diverse p97 functions and protein interactions are regulated by a large number of adaptor proteins. Alveolar soft part sarcoma locus (ASPL) is a unique adaptor protein that regulates p97 by disassembling functional p97 hexamers to smaller entities. An alternative mechanism to regulate the activity and interactions of p97 is by posttranslational modifications (PTMs). Although more than 140 PTMs have been identified in p97, only a handful of those have been described in detail. Here we present structural and biochemical data to explain how the p97-remodeling adaptor protein ASPL enables the metastasis promoting methyltransferase METTL21D to bind and trimethylate p97 at a single lysine side chain, which is deeply buried inside functional p97 hexamers. The crystal structure of a heterotrimeric p97:ASPL:METTL21D complex in the presence of cofactors ATP and S-adenosyl homocysteine reveals how structural remodeling by ASPL exposes the crucial lysine residue of p97 to facilitate its trimethylation by METTL21D. The structure also uncovers a role of the second region of homology (SRH) present in the first ATPase domain of p97 in binding of a modifying enzyme to the AAA+ ATPase. Investigation of this interaction in the human, fish, and plant reveals fine details on the mechanism and significance of p97 trimethylation by METTL21D across different organisms.

## Significance

Chaperones are key players in cellular homeostasis that can prevent potentially toxic protein misfolding and aggregation and allow stalled processes to resolve and continue. One of them is a AAA+ ATPase, p97, that acts as a molecular segregase in many processes in the cell. p97 can be regulated via adaptor proteins and posttranslational modifications. Here we present a structure that captures both regulatory mechanisms at once and explains how remodeling of p97 by the adaptor ASPL enables binding of the p97-modifying methyltransferase METTL21D.

p97, also known as CDC48 or valosin-containing protein (VCP), is a hexameric, ring-shaped AAA+ ATPase (ATPase associated with diverse cellular activities) that can extract ubiquitinated and nonubiquitinated substrate proteins from protein complexes and membranes (1, 2). The segregating activity of p97 is crucial in a number of cellular processes, such as membrane fusion, ER-associated protein degradation (ERAD), DNA repair, NF-κB activation, replication termination, and more (3–9). In cells, p97 is present predominantly as a stable hexamer, and each p97 monomer contains an N domain, followed by two nucleotide hydrolysis AAA+ modules, the D1 and D2 ATPase domains, and a disordered C-terminal tail (10, 11). Each AAA+ module contains three highly conserved motifs – the Walker A, Walker B, and the second region of homology (SRH) motif. Walker A and Walker B motifs mediate ATP binding and hydrolysis, respectively. The SRH motif is present in both D1 and D2 domains of p97, and it is conserved across species and related AAA+ ATPases (12). The two most prominent residues in the SRH motif are two arginines, Arg359 and Arg362 in the D1 domain, and Arg635 and Arg638 in the D2 domain of human p97. These arginines are located at the interface of p97 subunits, where they extend toward the nucleotide bound in the neighboring monomer, thereby modulating interprotomer communication and stabilizing a conformation of p97 necessary for cofactor binding (11, 13). The hexameric assembly of p97 is independent of the nucleotide-bound state and critical for the ATPase activity and segregase function (14).

The activity and interaction of p97 with numerous substrate proteins is mediated by adaptor proteins (9). ASPL (alveolar soft-part sarcoma locus; also known as UBXD9, TUG, or ASPSCR1) is a unique p97 adaptor protein that regulates p97 activity by disassembling the p97 hexamer into smaller oligomers (15, 16). ASPL utilizes its extended UBX (eUBX) domain with a conserved *cis*-Pro touch-turn motif to bind to the N domain of p97, and by forming a lariat structure through the UBX-domain extension, it disrupts the subunit interfaces in hexameric p97 (16). This results in the formation of a heterotetrameric complex containing two subunits of p97 and two molecules of ASPL. When overexpressed, ASPL causes accumulation of ubiquitinated substrates as an expected consequence of p97 inactivation and shifts the localization of p97 and ASPL to the nucleus (15). This renders ASPL a unique adaptor protein that impacts p97 structure, function, and subcellular localization at the same time. However, the exact role of the high-affinity p97:ASPL complex still remains to be explained.

In addition to p97 regulation via adaptor proteins, the activity and interactions of p97 are modulated by posttranslational modifications (PTMs). p97 is extensively modified by phosphorylation, acetylation, ubiquitination, and methylation; however, the modifying enzymes and the exact impact of those modifications are poorly understood (17). According to the PhosphoSitePlus database, 148 modifications are present in human p97 (18). Most of the PTMs were identified by high-throughput mass spectrometry, and only some of them have been additionally verified by other experimental methods. One example is trimethylation of p97 at residue Lys315, a modification introduced by METTL21D (also known as VCP-KMT or NVM-1). METTL21D is an S-adenosylmethionine (SAM)-dependent methyltransferase that has been shown to be up-regulated in human tumor tissues and to promote cancer cell metastasis (19–21).

The METTL21D-trimethylated Lys315 residue is deeply buried inside the p97 hexamer pore and inaccessible to methyltransferases (20). The trimethylation reaction was shown to be enhanced in the presence of ASPL; however, the detailed mechanism and biological relevance of this PTM remain unknown (19). To understand the mode of p97 methylation by METTL21D and the role of ASPL in this process, a structural investigation is required. Although separate structures of METTL21D, p97, and the p97:ASPL complex are available (16, 22), they cannot explain the exact mechanism of METTL21D binding and action on p97. Here, we report the crystal structures of a human p97:ASPL:METTL21D heterotrimeric complex and of the plant methyltransferase *At*METTL21D. Combined with in vitro and in vivo experiments, these structures reveal the determinants of METTL21D binding and trimethylation activity on p97 across species.

## Results

### METTL21D Binds the p97:ASPL Complex, but Not the p97 Hexamer.

**Fig. 1.**
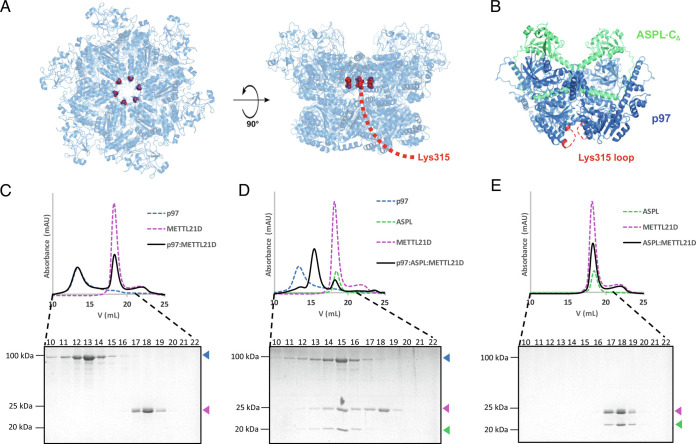
METTL21D binds to p97:ASPL, but not to hexameric p97. (*A*) Lys315 shown as red sphere is buried inside the narrow p97 hexamer pore (PDB ID: 5FTN). (*B*) In the crystal structure of the p97:ASPL heterotetramer (PDB ID: 5IFW), the loop harboring the Lys315 residue is disordered and exposed to the solvent, and Lys315 is accessible to METTL21D. (*C*–*E*) Interactions between p97, ASPL-C_Δ,_ and METTL21D analyzed by SEC; column fractions are analyzed by SDS–PAGE. (*C*) METTL21D does not bind to the p97 hexamer, and the two proteins elute separately from the SEC column. (*D*) METTL21D binds to p97 in the presence of ASPL-C_Δ_, resulting in a complex smaller than the p97 hexamer. (*E*) METTL21D does not bind to ASPL-C_Δ_. The proteins elute together due to similar molecular mass (21 kDa for ASPL-C_Δ_ and 25 kDa for METTL21D), but there is no shift indicating formation of a complex between these two proteins.

The starting hypothesis for this work was based on analysis of the published structures of full-length p97 and p97 bound to ASPL-C_Δ_, a truncated form of ASPL lacking the N-terminal UBL domain and a presumably unfolded polypeptide stretch linked to the eUBX domain (PDB ID: 5FTK and 5IFW) (16, 22). It was reported previously that METTL21D specifically trimethylates p97 at residue Lys315 (20). This residue is buried inside the narrow p97 hexamer pore and therefore inaccessible to a modifying enzyme (Fig. 1*A*). Subsequently, it was shown that the level of p97 trimethylation can be increased in the presence of ASPL (19). Analysis of the structure of the p97:ASPL-C_Δ_ heterotetramer complex (16) showed that the loop harboring Lys315 is flexible and exposed to the solvent (Fig. 1*B*). This led to the hypothesis that ASPL promotes p97-Lys315 trimethylation via p97 remodeling and exposure of the previously buried Lys315-carrying loop to METTL21D.

To test this hypothesis, the purified tag-free proteins were mixed in different combinations and subjected to size exclusion chromatography (SEC). First, we tested binding of METTL21D to the p97 hexamer in the absence of ASPL (Fig. 1*C*). METTL21D could not bind the hexameric form of full-length p97, and there was no complex formation, which is in agreement with previously published results (20). However, in the presence of the remodeling factor ASPL-C_Δ_, METTL21D could bind to full-length p97 and coeluted with both p97 and ASPL-C_Δ_ (Fig. 1*D*). The p97:ASPL-C_Δ_:METTL21D heterotrimeric complex eluted after the p97 hexamer peak, which indicates that binding of METTL21D in conjunction with ASPL-C_Δ_ retains p97 in a disassembled form. Additionally, METTL21D did not bind to ASPL-C_Δ_ in the absence of p97 (Fig. 1*E*), which suggests that ASPL-C_Δ_ enables the interaction between METTL21D and p97, but it does not directly contact METTL21D. Taken together, these results show that ASPL serves to mediate the interaction between full-length p97 and METTL21D without needing to interact with METTL21D itself.

### Crystal Structure of the p97-ND1:ASPL-C_Δ_:METTL21D Complex.

**Fig. 2.**
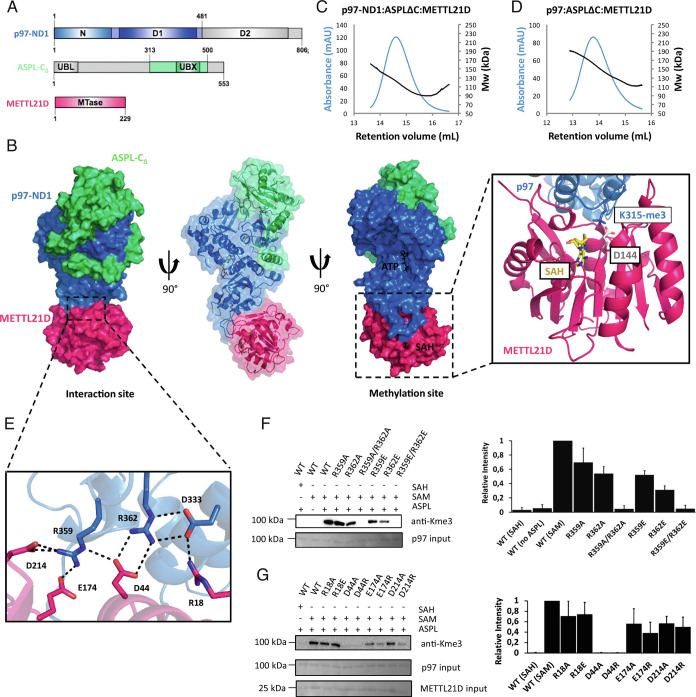
Crystal structure of the p97-ND1:ASPL-C_∆_:METTL21D complex. (*A*) Schematic representation of protein constructs used for crystallization (color-coded). (*B*) Crystal structure of the p97-ND1:ASPL-C_∆_:METTL21D complex rotated 90° stepwise with a close-up of the p97-ND1 K315 loop positioned on METTL21D. SAH, S-adenosyl-L-homocysteine. (*C*) SEC-RALS measurements for the truncated p97-ND1 fragment in complex with ASPL-C_∆_ and METTL21D. (*D*) SEC-RALS measurements for the full-length p97 in complex with ASPL-C_∆_ and METTL21D. (*E*) Magnified view into the interaction site between METTL21D and p97-ND1. Residues from the p97-D1 SRH motif, D333, R359, and R362, interact with highly conserved residues in METTL21D. (*F*) Mutation of the arginine residues, R359 and R362, from the p97-D1 SRH motif result in loss of trimethylation by METTL21D. (*G*) Mutation of the METTL21D residues interacting with p97 results in a reduced level of trimethylation, with the strongest effect of the D44A/R mutants, which completely lose their ability to trimethylate p97, as detected on the p97 samples by anti-Kme3. Signal intensities in *F* and *G* are quantitated in the *Side* panels.

To investigate the structural basis of the interaction between p97, ASPL, and METTL21D, the trimeric complex was purified and incubated with the METTL21D substrate S-adenosyl-methionine (SAM). Since the complex containing full-length p97 did not crystallize under tested conditions, the corresponding complex containing the truncated p97-ND1 fragment lacking the D2 ATPase domain was used. The trimeric complex of p97-ND1, ASPL-C_∆,_ and METTL21D (Fig. 2*A*) was crystallized in the presence of SAM, and X-ray diffraction data were acquired at a resolution of 3.0 Å (*SI Appendix*, Table S1).

The crystal structure of the complex contains one molecule each of p97-ND1, ASPL-C_∆,_ and METTL21D (Fig. 2*B*). In the active site of p97-ND1, a molecule of ATP is bound, whereas the active site of METTL21D contains a S-adenosyl-L-homocysteine (SAH) molecule (*SI Appendix*, Figs. S1*A* and Fig. 2*B*). The crystal structure has thus captured the product of the methylation reaction after Lys315 trimethylation and conversion of SAM to SAH. The most specific interactions between METTL21D and SAH arise from hydrogen bonds linking Asp96 to the ribose hydroxyl groups, and the stacking interaction of Trp126 with the adenine base of SAH (*SI Appendix*, Fig. S1*B*). The presence of ATP in the p97-D1 domain was somewhat unexpected, because we did not incubate the complex with ATP prior to crystallization. Possibly, the nucleotide came as an impurity from incubation with SAM. However, we could see during the refinement process that introducing ATP to the structural model was fitting the electron density better than adenosine diphosphate (ADP) (*SI Appendix*, Fig. S1 *C* and *D*). ATP binding to p97-ND1 in the complex with ASPL and METTL21D uses the same set of hydrogen bonds as ADP or ATPγS binding to hexameric p97 with the exception of the arginine finger. METTL21D is bound to the p97-D1 domain, and the p97 loop, which contains the target Lys315, positions its side chain toward the active site of METTL21D (Fig. 2*B*). To assess if the presence of the p97 D2 domain would interfere with METTL21D binding and Lys315 methylation, the p97-ND1:ASPL:METTL21D complex structure was compared with one subunit of the p97-ND1D2:ASPL heterotetrameric complex (PDB: 5IFW). In this structure, the D2 domain is in an orientation relative to the p97-ND1 moiety that would not obviously interfere with METTL21D binding and thus allow Lys315 methylation in full-length p97 (*SI Appendix*, Fig. S1*E*). In the crystal structure, Lys315 is in a trimethylated state, which could be validated by test refinements of all possible methylation states (*SI Appendix*, Fig. S1*F*). ASPL is bound on top of the p97-N domain, as described in previous work on the p97:ASPL-C_Δ_ complex, with the lariat structure of the ASPL-eUBX domain wrapped around the p97-N domain (16). There is no direct contact between METTL21D and ASPL, which is in agreement with analytical SEC results (Figs. 1*E* and 2*B*).

A striking result from the crystal structure is the presence of p97 in a monomeric state within the complex. The p97-ND1:ASPL–C_Δ_:METTL21D complex crystallized in space group P2_1_ with one trimer of the complex per asymmetric unit, and there is no indication from the crystal packing that a higher oligomer resembling either the p97 hexamer or the p97:ASPL-C_Δ_ heterotetramer may have formed (*SI Appendix*, Fig. S1*G*) as it was observed in previously published structures (16, 22). To verify the size of the heterotrimeric complex in solution, SEC-coupled right-angle light scattering (RALS) experiments were performed. The p97-ND1:ASPL–C_∆_:METTL21D complex exhibited light scattering as a particle of size between 90 kDa and 130 kDa, which corresponded to the size of the ~110-kDa heterotrimer (Fig. 2*C*). The complex containing the full-length ATPase, p97:ASPL-C_∆_:METTL21D, exhibited light scattering corresponding to a particle of size between 130 kDa and 170 kDa, indicating the expected trimeric complex of ~135 kDa (Fig. 2*D*). These results confirm that there is no higher oligomeric assembly than the 1:1:1 complex present in solution.

In the crystal structure, METTL21D is bound to the p97-D1 domain, sharing a large interaction surface of ~1,100 Å^2^. However, the crystallized trimeric complex contains a truncated form of p97 lacking the D2 domain, so we sought to verify if the structure indeed captures the main p97-METTL21D interaction. To test the strength of the interaction and compare the impact of the p97-D2 domain on complex stability, ITC measurements were performed (Table 1 and *SI Appendix*, Fig. S2). In the first experiment, METTL21D was titrated into the p97-ND1:ASPL-C_Δ_ complex containing the truncated form of p97. This revealed an unexpectedly high affinity of METTL21D for p97-ND1, with a K_d_ = 5.4 ± 0.7 nM, and an expected 1:1 molar ratio of binding (*SI Appendix*, Fig. S2*A*). When METTL21D was titrated to the p97:ASPL-C_Δ_ complex containing full-length p97, a comparable, only slightly lower affinity with a K_d_ = 14.4 ± 4.5 nM was revealed (*SI Appendix*, Fig. S2*B*). The somewhat lower affinity in this case may be explained by the presence of the D2 domain and the long, flexible D1–D2 linker region in full-length p97 that may influence the binding of METTL21D to a small extent. The titration of METTL21D to the monomeric D2 domain of p97 revealed an approximately 1,000-fold weaker interaction with a K_d_ = 4.3 ± 1.3 µM (*SI Appendix*, Fig. S2*C*). Together with the previous results, this suggests that the main interaction surface between p97 and METTL21D is the p97-D1 domain, as it is present in the crystal structure of the trimeric complex.

**Table 1.** ITC measurements (one-site binding model)

Cell	Syringe	N	K_D_ (M)
p97-ND1:ASPL-C_Δ_	METTL21D	1.02	(5.4 ± 0.7) × 10^−9^
p97:ASPL-C_Δ_	METTL21D	0.79	(14.4 ± 4.5) × 10^−9^
p97-D2	METTL21D	NA	(4.3 ± 1.3) × 10^−6^
p97^R359A/R362A^:ASPL-C_Δ_	METTL21D	NA	(7.5 ± 1.5) × 10^−6^
p97^R359E/R362E^:ASPL-C_Δ_	METTL21D	NA	no binding

### The p97-D1 SRH Motif Serves as a METTL21D Recognition Sequence.

After identifying the p97-D1 domain as the main interaction site for METTL21D, a more detailed analysis revealed a network of strong ionic interactions between p97 and METTL21D (Fig. 2*E*). The residues involved in the interaction belong to the highly conserved SRH motif of p97, including Arg359 that acts as the arginine finger crucial for nucleotide hydrolysis, and Arg362 that contributes to p97 hexamer stabilization (13). The main interaction residues find themselves approximately 17 Å (Cα^K315^–Cα^R362^) to 20 Å (Cα^K315^–Cα^R359^) away from the methylated Lys315, indicating that binding of METTL21D to p97 is independent of the following methylation reaction.

To verify this finding, we mutated the interacting residues in p97 and performed an analytical SEC screen to identify mutations that can abrogate the interaction with METTL21D. Single mutations of Arg359 and Arg362 to alanine had a weak effect on METTL21D binding, and the p97:ASPL:METTL21D complex could still be formed to some extent, as revealed by the shift of METTL21D on the gel to fractions containing higher molecular weight proteins (*SI Appendix*, Fig. S3 *A* and *B*). However, single mutations of Arg359 and Arg362 to an oppositely charged glutamate were sufficient to disrupt the interaction and, as expected, the double mutation of both arginine residues to glutamates had the same abolishing effect (*SI Appendix*, Fig. S3 *C*–*E*).

To quantify and further confirm the abrogating effect of the SRH motif mutations, we performed ITC measurements, where METTL21D was titrated to the previously purified p97^R359A/R362A^:ASPL-C_Δ_ and p97^R359E/R362E^:ASPL-C_Δ_ complexes. Both double mutations had a strong effect on METTL21D binding. In the presence of ASPL-C_Δ_, the p97^R359A/R362A^ mutant showed an approximately 500-fold reduction in binding affinity (K_d_ = 7.5 ± 1.5 µM; *SI Appendix*, Fig. S2*D*) compared with wild-type full-length p97 (K_d_ = 14.4 ± 4.5 nM, *SI Appendix*, Fig. S2*B*), and the p97^R359E/R362E^ mutation essentially completely abrogated the interaction with METTL21D (*SI Appendix*, Fig. S2*E*). The lack of binding is reflected in the inability of METTL21D to trimethylate the mutated p97^R359A/R362A^ and p97^R359E/R362E^ variants (Fig. 2*F*), suggesting that strong binding and correct positioning of METTL21D toward the p97 Lys315 side chain are essential for successful trimethylation. These results reveal a role of the SRH motif of the p97-D1 domain as a recognition sequence for METTL21D.

To confirm the p97-interacting residues of the enzyme as observed in the crystal structure, we mutated the four interacting residues, Arg18, Asp44, Glu174 and Asp214, of METTL21D to alanine or amino acids with opposite charge and tested the trimethylation activity on full-length p97 in the presence of ASPL-C_Δ_ (Fig. 2*G*). Single mutations of the interacting residues on the METTL21D side revealed strongest effect for residue Asp44, where the mutation causes complete loss of trimethylation. In conclusion, by disassembling the p97 hexamer, ASPL exposes the p97-D1 SRH motif that is otherwise buried at the monomer–monomer interface, allowing METTL21D to utilize it as its recognition site.

### The METTL21D Activity Is Required for Zebrafish Development.

Our structural and biochemical characterization revealed the formation of a heterotrimeric complex of p97:ASPL:METTL21D, the function of which is thus far unknown. To investigate the physiological significance of METTL21D and its ability to form complexes through the identified residues important for the interaction between METTL21D and p97, we deployed zebrafish (*Danio rerio*) as an experimental model.

**Fig. 3.**
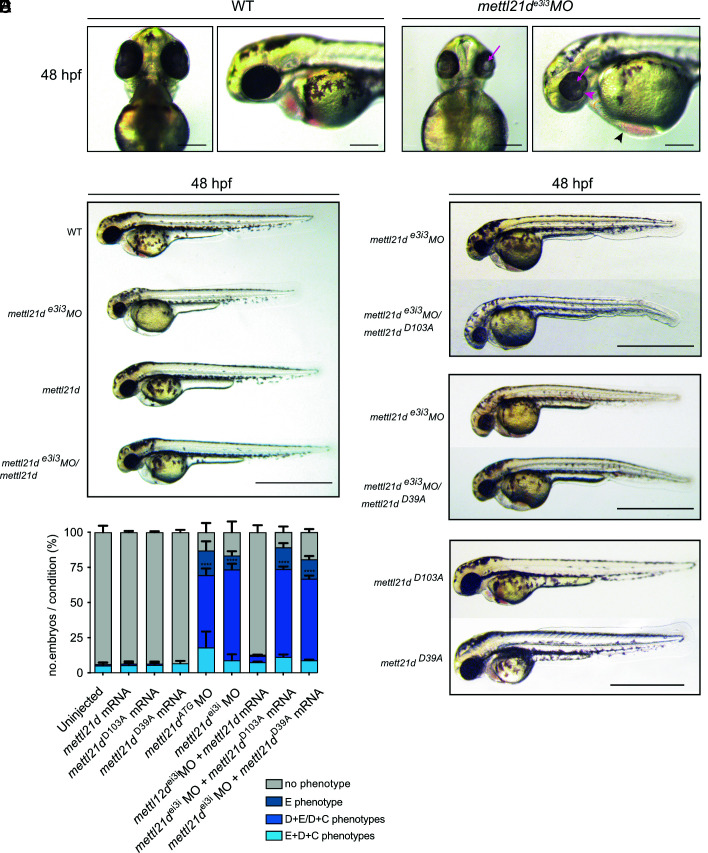
The disrupted function of METTL21D has an aberrant effect on zebrafish development. (*A*) Ventral and lateral views of zebrafish embryos with reduced *mettl21d* (*mettl21d^e3i3^MO*) levels appear to have an underdeveloped brain, a smaller eye with coloboma (red arrowhead) and defective lens formation (red arrow), and heart edema (black arrowhead) at 48 hpf when compared with uninjected wild-type (WT) embryos. (Scale bar = 200 mm.) (*B*) The phenotypes in a and a smaller body due to the lack of *mettl21d* (*mettl21d^e3i3^MO*) can be reversed by coinjecting the wild-type *mettl21d* mRNA (*mettl21d^e3i3^MO/mettl21d*), injection of which alone does not cause any observable phenotype (*mettl21d*). (*C*) Quantification of the aberrant phenotypes at 48 hpf shows that the wild-type mRNA can revert the phenotype, whereas the mRNA carrying the METTL21D activity mutation (D103A) and p97-interacting mutation (D39A) cannot. Embryos are categorized as “no phenotype,” “E phenotype,” “D + E/D + C phenotypes” and “E + D + C phenotypes;” E = cardiac edema, D = growth defects (small body and brain), C = lens defects and coloboma. N ≥ 3, n > 65 embryos per group; Percentage values are plotted as mean ± SD; n represents biologically independent samples over N independent experiments; Two-way ANOVA with Dunnett’s multiple comparison test; *P* < 0.05 considered significant. (*D*) Rescue experiment with the mRNA carrying a mutation for METTL21D activity (*mettl21d^e3i3^MO/mettl21d^D103A^*) fails to revert the phenotype to wild-type. (*E*) Rescue experiment with the mRNA carrying a mutation for p97 interaction (*mettl21d^e3i3^MO/mettl21d^D93A^*) fails to revert the phenotype to wild-type. (*F*) Control experiments showing the injection of the mRNAs carrying the D103A or D39A mutation in METTL21D (*mettl21d^D103A^* and *mettl21d^D93A^*, respectively) do not cause an aberrant phenotype in zebrafish embryos. All images in bde, *F* show lateral views of representative embryos at 48 hpf; Scale bar = 1 mm.

We first assessed the effects of METTL21D loss-of-function on zebrafish development using the transient morpholino (MO)-mediated knockdown technology at 48 h post fertilization (hpf). We used two different MOs, the translation start-site blocking MO and the MO blocking splicing at exon 3 of the zebrafish *mettl21d* gene, *mettl21d *^ATG^ and *mettl21d *^e3i3^, respectively. Reduced levels of *mettl21d* lead to clear and global developmental defects (Fig. 3 *A*–*C*). Specifically, the cardiac edema (Fig. 3*A*, black arrowhead) occurred alone or in combination with the growth defects with reduced body and brain size and/or with the eye defects including lens defects and coloboma (Fig. 3*A*, red arrow and red arrowhead, respectively). We classified the observed phenotypes into four different categories: no phenotype (the MO-injected embryos appear indistinguishable from wild-type embryos), E phenotype (cardiac edema only), D + E/D + C phenotypes (growth defect plus one other defect in eye or heart), and E + D + C phenotypes (all three phenotypes together) (Fig. 3*C*). The quantifiable and consistent developmental defects were observed when the uninjected wild-type embryos were compared with both MOs-injected embryos as noted in averages of 87% of *mettl21d *^ATG^ MO and 83.4% of *mettl21d *^e3i3^ MO embryos when all phenotypic categories combined (Fig. 3*C*).

For further experiments, we chose the splice-targeting *mettl21d *^e3i3^ MO, as it resulted in fewer severely affected embryos (9.9% vs. 17.5% in *mettl21d *^ATG^ MO injection, E + D + C phenotypes category) (Fig. 3*C*). To ensure that the described phenotypes were not due to off-target effects from MO injection, we cloned *mettl21d* mRNA and performed rescue experiments. Coinjection of *mettl21d *^e3i3^ MO and *mettl21d* mRNA reverted the growth and developmental phenotypes in average of 87.0% of all injected embryos (Fig. 3 *B* and *C*), indicating that the loss of METTL21D specifically leads to global growth and developmental defects.

To further discern the importance of differences between the general methylation activity of METTL21D and the potential p97:METTL21D interaction, we carried out rescue experiments with *mettl21d* mRNA encoding two different mutations. The first mRNA carried a D103A mutation at a catalytically important residue (D144A in humans) to verify whether the general methylation activity of METTL21D is necessary for embryonic development. Expectedly, coinjection of *mettl21d *^e3i3^ MO with this *mettl21d *^D103A^ mRNA was unable to rescue the knockdown defects with the average of 89% of all injected embryos having phenotypes (Fig. 3 *C* and *D*). This suggested that the global activity of METTL21D to methylate p97 and potentially other targets is necessary to establish normal development in zebrafish embryos. The second *mettl21d* mRNA encoded the D39A mutation (D44A in humans), which we showed to effectively abolish the interaction with p97 (Fig. 2*G*). Coinjection with this *mettl21d  *^D39A^ mRNA and *mettl21d *^e3i3^ MO failed to rescue the developmental defects with the average over 80% of embryos still having phenotypes (Fig. 3 *C*–*E*), indicating that this specific aa residue of METTL21D is necessary for development. Our data also suggest that the interaction of METTL21D with p97 or other potential targets and their subsequent methylation contribute to the biological roles of the methyltransferase. As a control, single injection of wild-type as well as mutated mRNAs did not induce any visible phenotypes (Fig. 3 *B*–*F*). Taken together, our data show that METTL21D is necessary for embryonic growth and development, and its methylation activity might be at least in part dependent on the specific interaction with p97.

### METTL21D Undergoes a Conformational Change upon Binding to p97.

Like other members of the distantly related group of METTL methyltransferases, METTL21D is a class-I methyltransferase adopting a classical Rossmann fold. The core fold of the enzyme contains seven β strands interrupted by α helices and is decorated with additional structural elements (*SI Appendix*, Fig. S4*A*). It contains conserved sequence motifs that are usually found in the METTL group of methyltransferases [motif 1, post 1, motif 2, motif 3, and the hallmark (D/E)XX(Y/F) motif] (23).

**Fig. 4.**
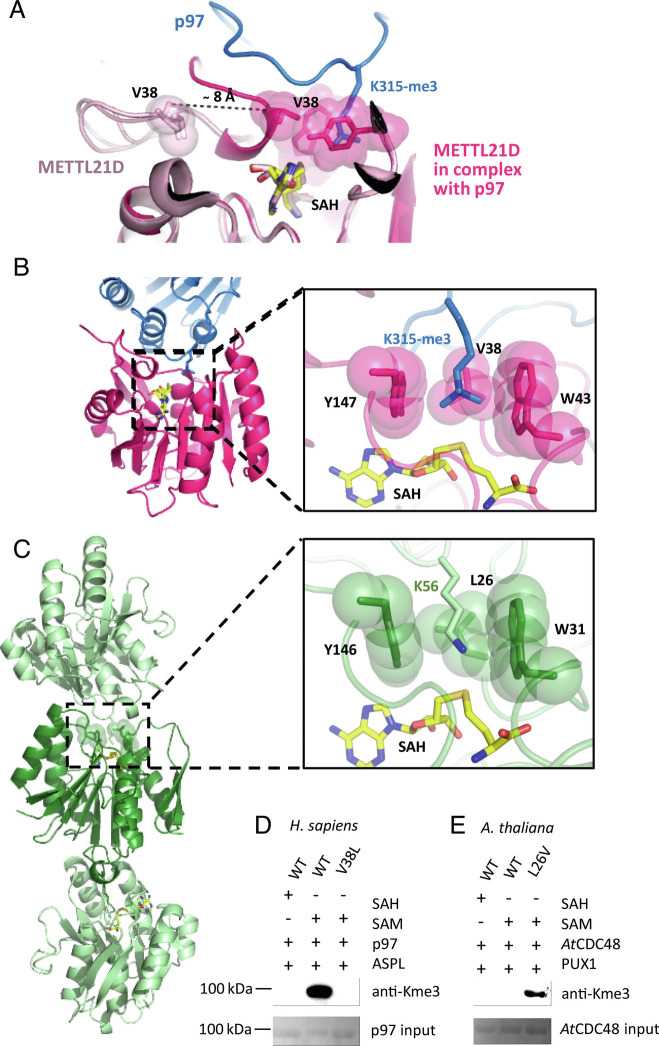
The hydrophobic cage of human and plant METTL21D. (*A*) METTL21D undergoes a conformational change upon binding to p97 and brings V38 closer to the active site. (*B*) The hydrophobic cage in human METTL21D is formed by valine, tryptophan, and tyrosine residues. (*C*) Crystal packing reveals METTL21D to be in an automethylation state. The packed *At*METTL21D molecules in the crystals are shown in dark and light green with K56 from a neighboring molecule (light green) pointing toward the active site of another *At*METTL21D molecule (dark green). K56 from the neighboring molecule points into the active site of *At*METTL21D, surrounded by a leucine, tryptophan, and tyrosine hydrophobic cage. (*D*) The hydrophobic cage mutation V38L mimicking the plant *At*METTL21D hydrophobic cage results in loss of trimethylation activity in human METTL21D. (*E*) The hydrophobic cage mutation L26V mimicking the human METTL21D hydrophobic cage results in gain of trimethylation activity in plant *At*METTL21D.

Comparison of the structure of monomeric human SAM-bound METTL21D (PDB ID: 4LG1) with METTL21D bound to p97-ND1:ASPL-C_Δ_ reveals a major difference in the position of the β2-α2 loop. Upon binding of METTL21D to p97 the β2 strand partially unfolds and forms a short α helix (Fig. 4*A*). Residue Val38 present in the newly formed α helix moves approximately 8 Å toward the modified Lys315 (Video S1). Together with residues Trp43 and Tyr147, Val38 forms a hydrophobic cage around the Lys315 side chain (Fig. 4*B*). Although the related methyltransferases display a high degree of structural similarity to METTL21D and contain a similar structure of the hydrophobic cage, they could not trimethylate p97, likely due to lack of the key residues for interaction with the p97-D1 SRH motif (*SI Appendix*, Fig. S4 *B*–*D*). Therefore, we decided to investigate the similarity of the interaction in another species, *Arabidopsis thaliana*.

### Mode of Action of METTL21D in Arabidopsis Thaliana Reveals the Role of the Hydrophobic Cage.

Since the p97-D1 SRH motif and the two key arginine residues, Arg359 and Arg362, are completely conserved at corresponding positions of protein homologs ranging from human to yeast (*SI Appendix*, Fig. S5*A*), we sought to investigate if p97 homologs in other organisms utilize the SRH motif for binding of a METTL21D homologous enzyme. The closest yeast homolog of METTL21D was previously shown to be unable to trimethylate CDC48 (20) and, in addition to that, we could not identify a disassembly factor similar to ASPL that could mediate exposure of the SRH motif for yeast methyltransferase binding. Therefore, we focused on the *Arabidopsis thaliana* orthologs, where we and others could previously demonstrate that the plant protein PUX1 disassembles the plant AAA+ ATPase *At*CDC48 in a similar manner as ASPL disrupts the p97 hexamer (24, 25) and a homologous enzyme to METTL21D could be identified (19).

To our surprise, *At*METTL21D was unable to trimethylate *At*CDC48 (*SI Appendix*, Fig. S5*B*). This could be explained by apparently weaker binding of the plant enzyme to *At*CDC48 in the presence of PUX1 as revealed by SEC experiments (*SI Appendix*, Fig. S5*C*). However, *At*METTL21D contained two necessary interaction residues (*SI Appendix*, Fig. S6) and based on the observation that human METTL21D with mutations in single-interaction residues other than Asp44 could still trimethylate p97 (Fig. 2*G*), we speculated that there must be an additional reason for the lack of *At*CDC48 trimethylation by *At*METTL21D. Since the *At*CDC48:PUX1:*At*METTL21D complex did not crystallize, we analyzed the structure of *At*METTL21D to gain insight into its mode of action (*SI Appendix*, Fig. S5 *D* and *E*). The crystal structure showed *At*METTL21D in a premethylation state and crystal packing of *At*METTL21D suggested an automethylation mode with residue Lys56 from one molecule pointing toward the active site of the neighboring molecule (Fig. 4*C*). This lattice interaction was insightful for two reasons. First, it revealed that the lysine residue pointing to the binding pocket was indeed not trimethylated. However, when we probed our *At*CDC48 samples with an anti-monomethyl-lysine antibody, we observed that *At*METTL21D monomethylated itself, as well as *At*CDC48 in the presence of PUX1 (*SI Appendix*, Fig. S5*F*). Additionally, the plant mutant corresponding to the human SRH-motif mutant, *At*CDC48^R362E/R365E^, abrogated monomethylation by *At*METTL21D, suggesting that *At*METTL21D monomethylates *At*CDC48 in a similar manner as human METTL21D methylates p97. Secondly, the structure revealed a difference in the hydrophobic cage that accommodates the methylated ε-amino group. Instead of a *Val*-Trp-Tyr cage (Fig. 4*B*), the plant enzyme contains a *Leu*-Trp-Tyr cage (Fig. 4*C*).

We hypothesized that substitution of valine by the larger leucine residue introduces steric hindrance on the possible methylation level. To test this hypothesis, we mutated the hydrophobic cage of plant and human METTL21D to mimic one another. Indeed, substitution of valine with leucine in the human METTL21D enzyme resulted in loss of trimethylation activity (*SI Appendix*, Fig. S5*D*). Subsequently, substitution of leucine with valine in plant *At*METTL21D resulted in gain of trimethylation activity (*SI Appendix*, Fig. S5*E*). The signal was rather weak, likely due to the weaker binding of *At*METTL21D to its target *At*CDC48, but nevertheless detectable by the pan-Nε-trimethyl antibody (referred to as anti-Kme3), proving that the hydrophobic cage can be the determining factor of a methyltransferase ability to mono-, di- or trimethylate. Therefore, we suggest that the leucine to valine substitution in the hydrophobic cage of *A. thaliana* METTL21D acts as a switch that shifts the methyltransferase activity from monomethylation to trimethylation.

## Discussion

The AAA+ ATPase p97 and its adaptor proteins are important players in a number of biological processes essential for cellular function and viability. Understanding the regulation of p97 through adaptor proteins and PTMs is especially relevant in the context of cancer and neuromuscular disorders, since aberrant function of p97 and its interacting partners have been shown to play a significant role in cancer metastasis and diseases such as amyotrophic lateral sclerosis (ALS) and inclusion body myopathy with early-onset Paget disease and frontotemporal dementia (IBMPFD) (26).

In this study we present the crystal structure of a Lys315 trimethylated p97:ASPL:METTL21D complex and the structure of the plant enzyme *At*METTL21D in the premethylation state. We elucidated the detailed binding mode of METTL21D to p97, and explained the role of ASPL in exposing both the binding and methylation site in p97. Furthermore, we verified the relevance of identified interacting amino acids in vitro and in vivo, showing a strong effect of a *METTL21D* knockdown in zebrafish embryo development. Finally, the structural comparison of plant *At*METTL21D to its human ortholog revealed an important difference in the architecture of the hydrophobic cage that accommodates the modified lysine side chain, showing that the hydrophobic cage can be the determining factor in methylation level capacity of the methyltransferase.

Previous studies showed that ASPL and its *A. thaliana* homolog, PUX1, can disassemble their cognate hexameric AAA+ ATPases, p97 and *At*CDC48, to smaller species (15, 24, 27). Together with earlier detailed structural investigations of the p97:ASPL and the *At*CDC48:PUX1 complexes (16, 25), the mechanism of the disassembly process was partly elucidated; however, its purpose remained unclear. In this study, we could show that ASPL-induced p97 disassembly is indispensable for promoting METTL21D binding to p97 and subsequent trimethylation of the target residue, Lys315. Additionally, p97 disassembly potentially allows other PTMs to be introduced. Indeed, other mapped p97 modification sites, such as Ser326, phosphorylated by ATM/ATR (28), and Ser352 and Ser746, phosphorylated by Akt (29, 30), are also accessible only when p97 is in its nonhexameric, disassembled form.

The interaction between p97 and METTL21D reveals two important points. First, our study demonstrates that the p97-D1 SRH motif not only serves its well-established role in promoting p97 hexamer stability and stimulating ATPase activity, but that it can also serve as a docking site for interaction partners. The p97-D1 SRH motif is located within the wider 80-amino acid (aa) sequence that was previously identified to be important for binding of METTL21D (20). The SRH motif consensus sequence for the AAA+ family of ATPases was determined to be a 19-aa sequence, X(T/S)(N/S)X_5_DXAX_2_RX_2_RX(D/E) (31), showing that the crucial arginine-finger residue Arg359 of p97 and the downstream Arg362 within the 4-aa motif (RXXR) are completely conserved within the family. Interestingly, the p97-D2 domain contains a proline residue within this motif (RPGR), which is shared by other closely related AAA+ ATPases, and we could show that METTL21D binds only very weakly to the p97-D2 domain, despite the high structural similarity to p97-D1. In addition to the difference in the recognition motif, many related AAA+ ATPases form oligomers where the corresponding lysine residue would be buried as in hexameric p97, but there are no known disassembly factors. One example is the AAA+ ATPase NSF, which forms a stable hexamer and uses its pore to disassemble the SNARE complex after vesicle fusion (8, 32). On the contrary, there are examples of AAA+ ATPases that do not form oligomeric structures or are not as stable as the p97 hexamer, such as VPS4 (33) and katanin (34). These ATPases exist in a monomer–oligomer equilibrium that could facilitate an interaction or modification similar to the one introduced by METTL21D.

Utilization of the p97-D1 SRH motif with phenylalanine in the second position (RFGR) as a recognition sequence for METTL21D is an exceptionally selective way of preventing unspecific binding to related AAA+ ATPases. In addition to that, it seems unlikely that the interaction site will accumulate mutations, since the SRH motif is evolutionarily highly conserved, both across species and other AAA+ ATPases, and indispensable for p97 ATP hydrolysis. None of the disease mutations in p97 map onto this region, since they would most likely result in a lethal phenotype. This, together with the fact that most of p97 is found to be trimethylated at Lys315 in human cells (20), supports the hypothesis that Lys315me3 might be a permanent modification that contributes to the physiological state of p97 in vivo to ensure optimal p97 activity.

Second, our complex structure shows that in certain cases, the interaction site between the methyltransferase and the target protein may be located outside the peptide sequence surrounding the methylation site. Much of the current knowledge on protein methylation is based on histone H3 and H4 methylation, where the flexible N-terminal tails of histones are frequently methylated, resulting in changes in gene expression (35). In this case, there are often additional factors such as DNA-binding proteins, lncRNAs, or ncRNAs that recruit the methyltransferase or mediate the interaction between the histone peptide and the histone methyltransferase (36). However, nonhistone methyltransferases that methylate specific targets are likely to interact differently with their substrates, and even though they may share a high degree of structural similarity as in the family comprising METTL21A-D, single residues far away from the active site can discriminate between their targets. Indeed, when residues around Lys315 in p97 are mutated to resemble the histone H3 sequences, the methyltransferase still retains the ability to trimethylate the lysine residue (37). The authors claim that this is due to a more relaxed sequence specificity of METTL21D around the target p97-Lys315. We, however, propose that the presence of the unchanged and spatially distant interaction motif along with a flexible loop that carries the lysine residue serve as determining factors for the ability of METTL21D to modify p97 independent of the residues proximal to the target Lys315.

Even though our results strongly suggest that ASPL is required for accessibility of the D1-SRH motif and the Lys315-carrying loop of p97, we cannot completely exclude the possibility that the trimethylation by METTL21D might occur cotranslationally. METLL21D might indeed bind to p97 monomers prior to their assembly into a hexamer. However, the general binding mode would still have to remain the same, where the emerging ND1 domain of p97 would have to be folded to allow for METTL21D binding to the SRH motif and positioning of the active site for methylation of Lys315. A study (19) that supports the involvement of ASPL in METTL21D-induced trimethylation of p97 has also shown a direct interaction between METTL21D and ASPL, in particular the C-terminal part of ASPL (aa 280 to 553). With our truncated fragment (aa 313 to 500), we do not observe an interaction between these two proteins, neither in the structure nor in SEC experiments. It is likely that the interaction motif in ASPL is located exactly at amino acids 280 to 312 or 501 to 553. It would be interesting to know if these sequences contribute to recruitment of METTL21D to p97 in cells. Our findings, however, show that a direct interaction between METTL21D and ASPL is not a prerequisite for recruitment of METTL21D to p97 in vitro.

The in vivo experiments in zebrafish confirmed the specificity of the p97–METTL21D interaction and demonstrated the effect on zebrafish embryonic development. Although we used this experiment primarily to test the general structure–function relationship, the lack of a properly formed brain, eye, heart, and jaw points to a potentially aberrant effect in cell survival, growth, or migration during development. Cell migration is not only crucial at different stages of embryonic development, but it is also an important factor in cancer metastasis (38). Previous studies have shown that METTL21D plays a role in cell migration during cancer (21), and we speculate that the methylation of p97 could affect the process of cell migration in different cellular scenarios and developmental stages. Indeed, it has been shown recently that knockdown of p97 results in accumulation of ubiquitinated RhoA, subsequently leading to stabilization of actin K and reduced cell motility (39). However, further research is necessary to confirm the exact physiological role of METTL21D and trimethylated p97 in zebrafish development.

The structural analysis of *Arabidopsis thaliana* METTL21D revealed an interesting difference in the hydrophobic cage around the target lysine side chain compared with the human METTL21D. METTL21D not only contains a hydrophobic cage that can be found in other related methyltransferases, but also resembles the hydrophobic cage of histone trimethylation mark “reader” proteins, such as the bromodomain PHD finger transcription factor (BPTF) (40). The occurrence of a hydrophobic cage, or at least a pair of aromatic residues, appears to be a common way of ensuring subsequent methylation reactions to occur, as in the example of another SAM-dependent bacterial methyltransferase, PrmA (41). However, in the case of PrmA, the orientation of the ribosomal target protein L11 and its flexibility impact the level of methylation, whereas in the case of METTL21D, it seems that proper orientation toward the substrate is required, but that the hydrophobic cage can determine the level of methylation via steric constraints.

**Fig. 5.**
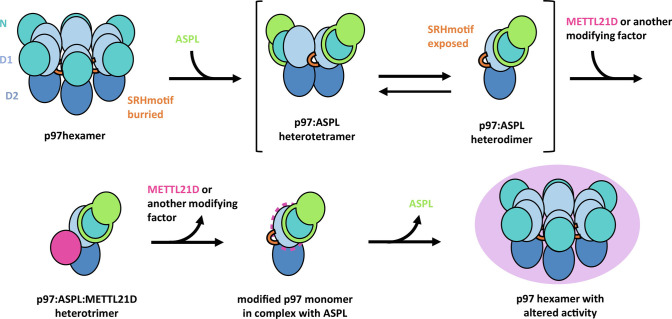
Mechanistic model of ASPL-mediated p97 methylation by METTL21D. ASPL promotes PTMs by exposing new interaction surfaces of p97. Upon binding of ASPL, hexameric p97 disassembles to smaller oligomers, and the SRH motif becomes exposed. This enables binding of METTL21D (and possibly other modification factors) to the SRH motif of p97 and modification of the previously inaccessible residue K315. After detachment of METTL21D (or another enzyme) and ASPL, modified p97 may reassemble to a hexamer with modified unfolding activity.

In summary, the crystal structure of the p97-ND1:ASPL-C_Δ_:METTL21D complex shows a way of protein binding to p97 by utilization of the D1-SRH motif, which is usually buried at the p97 monomer–monomer interface (Fig. 5). Upon disruption of the hexamer new interaction surfaces as well as target residues are exposed to novel binding partners and modifying enzymes. We propose that after release of ASPL and METTL21D via a so far undetermined mechanism, modified p97 monomers reassemble into a hexamer with altered properties as recently shown (42). By involving a UBX protein and a modifying enzyme, this molecular mechanism serves to integrate the two major modes of p97 regulation, by adaptor proteins and through PTM. Further research will be needed to elucidate the mechanism of introduction and impact of PTMs on the activity of p97 and related molecular machines.

## Materials and Methods

### Protein Expression and Purification.

Plasmids carrying the DNA for wild-type p97, ASPL, and human MTases were transformed into *E. coli* Rosetta 2 (DE3) T1^R^ and the p97 mutants and METTL21D mutants into *E. coli* BL21 (DE3) pLysS. A large-scale culture was grown at 37 °C to optical density 0.8. Gene expression was induced with 0.5 mM isopropyl-β-D-thio-galactopyranoside (IPTG) at 16 °C overnight. Human p97, plant *At*CDC48, and human ASPL-C_∆_ (residues 313 to 500) were purified as previously described (16, 25). Protein was loaded on a Superdex S200 16/60 column (GE Healthcare) in SEC Buffer A in case of p97 and *At*CDC48 (20 mM HEPES pH 8.0, 200 mM NaCl, 5 mM MgCl_2_) or SEC Buffer B in case of ASPL-C_∆_ (20 mM HEPES pH 8.0, 200 mM NaCl, 2 mM DTT). Fractions containing pure protein were pooled and concentrated, flash-frozen in liquid nitrogen, and stored at −80 °C.

Human METTL21D was expressed and purified as a polyhistidine-tagged fusion protein (Addgene plasmid #60092). The bacterial pellet was thawed and resuspended in lysis buffer (PBS, 5% glycerol, 300 mM NaCl, 0.1% Triton-X-100, 0.5 mM DTT) containing protease inhibitors and DNase. Bacterial cells were disrupted by homogenization, and the lysate was centrifuged at 20,000 *g* for 45 min. The supernatant was loaded on Ni-NTA resin (Qiagen) and purified as described for *At*CDC48 (25). The polyhistidine tag was cleaved during overnight dialysis by TEV protease. After removing the TEV protease on a second Ni-NTA column, the protein was concentrated and loaded on a Superdex S200 16/60 column (GE Healthcare) in SEC Buffer B. Fractions containing pure METTL21D were pooled and concentrated. Plant *At*METTL21D was purified using the same protocol, with the exception of a higher salt concentration used in buffers (500 mM NaCl) to prevent aggregation. Additionally, a cysteine to serine mutation, C4S, was introduced in *At*METTL21D to avoid formation of disulfide bonds during crystallization.

### Removal of Prebound SAH/SAM from METTL21D.

To remove prebound SAH and SAM from METTL21D, the enzyme was unfolded and refolded. Previously purified protein was diluted in SEC buffer B to a concentration of 1 mg/mL and dialyzed in the same buffer containing 0.5 M guanidinium chloride overnight at 4 °C. Next day, the protein was dialyzed in SEC buffer B for 6 h at 4 °C. For every step, a sample was taken for subsequent analysis on HPLC. After refolding, the protein was concentrated and loaded on a Superdex S200 16/60 column. Fractions containing the refolded protein were pooled and concentrated, flash-frozen in liquid nitrogen, and stored at −80 °C.

### Assembly of the p97:ASPL:METTL21D Complex.

To assemble the heterotrimeric p97-ND1:ASPL-C_∆_:METTL21D complex, first the complex of p97-ND1 and ASPL-C_Δ_ was purified by SEC as described previously (16). After concentrating the purified heterodimeric complex, METTL21D and the cofactor SAM were added to the purified p97-ND1:ASPL-C_∆_ complex and incubated on ice for 1 h. This mixture was subjected to an additional SEC on the Superdex S200 16/60 column in SEC Buffer B, and fractions containing all three proteins were pooled, concentrated to 10 mg/mL, flash-frozen in liquid nitrogen, and stored at −80 °C.

### Protein Crystallization and Structure Determination.

The p97-ND1:ASPL-C_∆_:METTL21D complex was screened for crystallization by mixing equal volumes (200 nL) of the protein complex and reservoir solution using the sitting-drop vapor diffusion method at 4 °C in 96-well plates and commercial crystallization screens. Crystals of the p97-ND1:ASPL-C_∆_:METTL21D complex appeared after 2 d in a condition containing 16% PEG 8000, 0.2 M calcium acetate, and 0.1 M MES (pH 6.5). The *At*METTL21D crystals appeared after 6 d in a condition containing 10% w/v ethylene glycol, 0.1 M HEPES (pH 7.5), and 10% w/v PEG 8000. All crystals were cryoprotected with 20% ethylene glycol and flash-frozen in liquid nitrogen.

Diffraction images were collected on BL14.1 at BESSY II (Helmholtz-Zentrum Berlin, Germany) and processed with XDS (43). The crystals belonged to the monoclinic space group P2_1_. Phases for the p97-ND1:ASPL-C_∆_:METTL21D complex were obtained by molecular replacement, using the previously analyzed structure of the p97-ND1:ASPL-C_∆_ (PDB ID: 5IFS) as search model. The structure of p97-ND1:ASPL-C_∆_:METTL21D was manually built using COOT (44, 45) and refined with PHENIX (46) in an iterative manner until structure factor amplitudes derived from the built model fitted the experimentally observed diffraction pattern, reflected in the R_work_ and R_free_ values in a range expected for the given resolution. Structures were validated using MolProbity (47) and the PDB validation server. Structure analysis, superpositions, and figures were done in PyMol. X-ray diffraction data and structure models for *At*METTL21D and p97-ND1:ASPL-C_Δ_:METTL21D are available from the Protein Data Bank under entry codes 7OAS and 7OAT refs. 48 and 49.

### Methylation Assay and Western Blot.

For the methylation assay, proteins were mixed in equimolar concentrations (10 µM) and incubated for 1 h at room temperature (RT) in the reaction buffer (20 mM HEPES pH 8.0, 200 mM NaCl) in the presence of SAM or SAH as negative control. After the proteins were separated by SDS–PAGE, with the Precision Plus Protein™ Dual Color Standards (Biorad) as molecular weight marker, the proteins were transferred on a nitrocellulose membrane in wet transfer buffer (25 mM Tris/HCl, pH 8.0, 192 mM glycine, 20% methanol, 0.04% SDS) for 1 h at 400 V. Afterward, the membrane was blocked for 1 h in 3% skimmed milk in TBST buffer (50 mM Tris/HCl, pH 8.0, 150 mM NaCl, 0.1% Tween 20) at RT. The blocking solution was discarded, and the primary antibody was added to 10 mL antibody incubation buffer in a dilution recommended by the manufacturer and incubated overnight at 4 °C. Next day, the membrane was washed three times for 10 min with TBST buffer at RT. The primary antibody was added to 10 mL antibody incubation buffer in a dilution recommended by the manufacturer and incubated for 1 h at RT. The Abcam antibody ab76118 was used for detection of lysine trimethylation and the Abcam antibody ab23366 for recognition of monomethylation. This incubation was followed by three washing steps. Finally, a two-component HRP substrate (Pierce ECL western blotting substrate) was used to detect the secondary antibody (HRP-linked anti-rabbit IgG, CST #7074). The membranes were imaged by the LAS 4000 documentation system in a setting appropriate for secondary antibody detection.

### Analytical Size Exclusion Chromatography (aSEC).

Binding of p97 mutants in complex with ASPL-C_Δ_ to wild-type METTL21D was investigated using a Superose 6 10/300 column (GE Healthcare). The proteins were mixed together, incubated at 4 °C for 1 h and loaded on the column. Fractions were collected and analyzed by SDS–PAGE.

### Isothermal Titration Calorimetry (ITC).

ITC experiments were performed using a VP-ITC titration microcalorimeter (GE Healthcare). All titrations were performed in a buffer containing 20 mM HEPES/NaOH pH 8.0, 200 mM NaCl at 18 °C. 80 µM of METTL21D was titrated into 8 µM wild-type or mutant full-length or truncated p97 in complex with ASPL-C_∆_ (the exception being the D2 domain of p97 that already is a monomer, so ASPL-C_Δ_ was not added). Raw data were fitted by nonlinear least squares using a one-site binding model in the ORIGIN7 software.

### RALS Coupled to SEC.

To determine the oligomeric state and absolute molecular mass of the protein complex in solution, a RALS and refractive index (RI) detector were coupled to a Superdex S200 10/300 SEC column. The column and detectors were equilibrated with the appropriate gel-filtration buffer, and 100 μL 3 mg/mL of complex was loaded on the column. The results containing the UV, RALS and RI signal were assessed with OmniSEC software.

### Zebrafish husbandry.

Zebrafish were bred, raised, and maintained in accordance with the guidelines of the Max Delbrück Center for Molecular Medicine and the local authority for animal protection (Landesamt für Gesundheit und Soziales, Berlin, Germany). Zebrafish were maintained under continuous water flow and filtration with automatic control for a 14:10-h light/dark cycle at 28.5 °C. Fertilized eggs were collected and raised under standard laboratory conditions [at 28.5 °C in E3 solution (5 mM NaCl, 0.17 mM KCl, 0.33 mM CaCl_2_, 0.33 mM MgSO_4_, pH 7.4)]. The AB/TL wild-type line was used in this study.

### cDNA Construction and mRNA Synthesis.

The closest zebrafish homolog to human METTL21D was determined by sequence alignment (Uniprot ID: X1WE47). The corresponding synthetic zebrafish *mettl21d* cDNA was purchased (Eurofins Genomics) and cloned into the pET28a vector. To synthesize the corresponding mRNAs, in vitro transcription was done using the T7 MAXIscript® Kit (Thermo Fisher Scientific) from linearized plasmid DNA template at 37 °C. To increase transcript stability and translation efficiency, the mRNA was polyadenylated using the Poly(A) Tailing Kit (Thermo Fisher Scientific) and purified with the RNeasy Mini Kit (Qiagen) before injection.

### Microinjection.

MOs were dissolved in RNase/DNase-free water to make 1 mM stock solutions and used at a 1:2 dilution. Diluted MO solutions were incubated at 65 °C for 10 min before injection into the yolk of 1 to 4 cell stage embryos. All MO used were obtained from GeneTools. Capped mRNA (100 pg/μL each) was injected into one-cell stage embryos for both single and coinjection experiments. Injection volume was 1 nL per embryo for all experiments.

### Phenotypic Analysis.

The phenotypic analysis of wild-type (AB/TL) embryos and respective MO- and/or mRNA-injected embryos was performed at 48 hpf. The observed phenotypes were categorized into four phenotypic groups: no phenotype (the MO-injected embryos appear indistinguishable from wild-type embryos), E phenotype (cardiac edema only), D + E/D + C phenotypes (growth defect plus one other defect in eye or heart), and E + D + C phenotypes (all three phenotypes together). Each experiment was performed as three independent replicates (N = 3) with respective controls. The data for WT and *mettl21d^e3i3^* MO-injected embryos were analyzed as an aggregate of N = 12 and N = 9 independent experiments, respectively. Altogether n = 1,081 WT, n = 869 *mettl21d ^e3i3^* MO-injected, n = 308 *mettl21d ^ATG^* MO-injected, n = 247 *mettl21d*-injected, n = 219 *mettl21d*^D103A^-injected, n = 225 *mettl21d*^D39A^-injected, n = 331 *mettl21d^e3i3^* MO*/mettl21d*-injected, n = 336 *mettl21d^e3i3^* MO*/mettl21d*^D103A^-injected, and n = 326 *mettl21d^e3i3^* MO*/mettl21d*^D39A^-injected embryos were analyzed.

## Supplementary Material

Appendix 01 (PDF)

Movie S1. **Movement of the METTL21D Val38 residue upon binding of METTL21D to p97.** Upon binding of METTL21D (magenta) to p97 (blue) the METTL21D residue Val38 (represented as green spheres) moves approximately 8 Å towards the tri-methylated Lys315. This movement of Val38 results in closing of the hydrophobic cage around Lys315, together with residues Trp43 and Phe147 (upper and lower residues depicted as spheres in magenta, respectively). The crystal structure of the METTL21D monomer (PDB: 4LG1) was used as starting structure of unbound METTL21D.

## Data Availability

The electron density maps and corresponding molecular models have been deposited to the Protein Data Bank (PDB) under accession codes 7OAT for the p97:ASPL:METTL21D structure (48) and 7OAS for the AtMETTL21D structure (49).
