# Supplementary material for: Structural remodeling of AAA+ ATPase p97 by adaptor protein ASPL facilitates posttranslational methylation by METTL21D
Source: Proc Natl Acad Sci U S A. 2023 Jan 19;120(4):e2208941120. doi: 10.1073/pnas.2208941120 (PMC9942839; doi:10.1073/pnas.2208941120)
Supplement: Supplementary file 1 — Appendix 01 (PDF) [file pnas.2208941120.sapp.pdf]

Supplementary Information for

Structural remodeling of AAA+ ATPase p97 by adaptor protein ASPL facilitates post-translational methylation by METTL21D

Saša Petrović[1,2,3*], Yvette Roske[1], Biria Rami[2], Mai Hoang Quynh Phan[1,4], Daniela Panáková[1], Udo Heinemann[1,2*]

[1] Max Delbrück Center for Molecular Medicine, Robert-Rössle-Straße 10, 13125 Berlin, Germany

[2] Institute for Chemistry and Biochemistry, Freie Universität Berlin, Takustraße 6, 14195 Berlin, Germany

[3] Current address: Institute for Biochemistry and Biology, Universität Potsdam, Karl-Liebknecht-Straße 24-25, 14476 Potsdam, Germany

[4] Current address: Max Planck Institute for Molecular Genetics, Ihnestraße 63-73, 14195 Berlin, Germany

***Corresponding authors:** Saša Petrović and Udo Heinemann

Email: sasa.petrovic@uni-potsdam.de, heinemann@mdc-berlin.de

This PDF file includes:

Supplementary Table 1
Supplementary Figures S1 to S6

Other supplementary materials for this manuscript include the following:

Movie S1

Supplementary Table 1.

**Data collection statistics**	**p97-ND1:ASPL-C$_{\Delta}$:METTL21D**<br>pre-incubated with SAM	***At*METTL21D**<br>pre-incubated with SAM
Beamline	BESSY II 14.1	BESSY II 14.1
Wavelength (Å)	0.9184	0.9184
Temperature (K)	100	100
Space group	$P2_1$	$P4_12_12$
Unit cell		
a, b, c (Å)	54.3, 69.6, 140.2	58.3, 58.3, 142.8
α, β, γ (°)	90.0, 94.4, 90.0	90.0, 90.0, 90.0
Resolution* (Å)	49.3 – 3.00 (3.15 – 3.00)	45.16 – 1.82 (1.93 – 1.82)
No. of observed reflections	128,484 (17,427)	171,458 (26,723)
No. of unique reflections	20,828 (3,167)	22,897 (3,628)
R_{meas} (%)	27.3 (198.3)	10.3 (389.0)
< I/σ(I) >	6.65 (0.79)	13.36 (0.66)
$CC_{1/2}$ (%)	98.6 (32.4)	99.9 (52.8)
Completeness (%)	98.1 (92.7)	99.7 (99.7)
**Refinement statistics**		
R_{work} / R_{free}	22.5 /27.0	19.7 / 23.8
No. of atoms	6,562	1,910
Protein	6,415	1,710
Ligands	89	29
Solvent (water, ions)	58	171
RMSD		
Bond lengths (Å)	0.006	0.007
Bond angles (°)	0.40	0.92
Ramachandran statistics		
Most favored (%)	95.5	98.1
Allowed (%)	4.1	1.9
Disallowed (%)	0.4	0
Average B-factors (Å^2)	78.66	54.71

Numbers in parentheses always refer to the outer resolution shell.

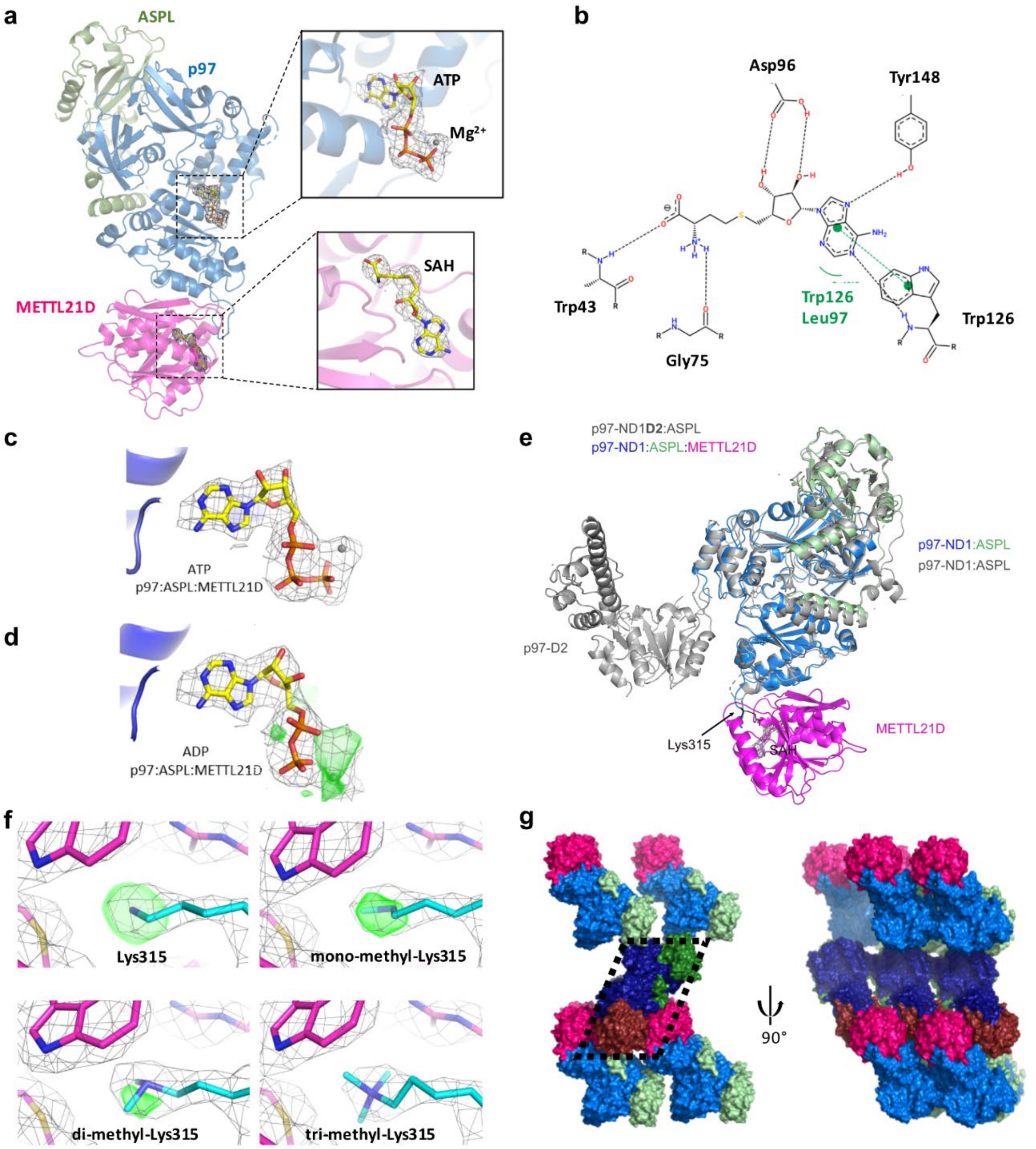

Supplementary Figure 1. Analysis of the p97:ASPL:METTL21D crystal structure. a, Position of ligands in the structure with electron density for ATP, Mg^{2+} and SAH. The 2Fo-Fc map is contoured at 1σ level and 2 Å distance from selected atoms. **b,** METTL21D residues involved in SAH binding. **c, d,** Validation of nucleotide binding in disassembled p97 complex structures. ADP/ATP molecules are shown as stick model bound in the p97:ASPL:METTL21D complex structures depicted with 2Fo-Fc electron density represented as gray mesh at a 1σ contour level. Fo-Fc difference electron densities are contoured at 3σ and highlighted in green. Bound magnesium ions are shown as gray spheres. **c,** shows an ATP nucleotide bound in the heterotrimeric p97:ASPL:METTL21D complex, whereas **d,** presents a modeled ADP molecule

instead of ATP for comparison in the same complex. Obvious green difference electron density together with bulky 2Fo-Fc density in **d,** suggests the probability for a third bound phosphate as present in ATP. **e,** Comparison of p97-ND1:ASPL:METTL21D and p97-ND1D2:ASPL complexes. Superposition of one subunit of the heterotetrameric p97ND1D2:ASPL complex (p97-ND1:ASPL shown in light grey color) onto the trimeric p97ND1:ASPL:METTL21D complex (p97-ND1:ASPL depicted in blue and green respectively, METTL21D depicted in magenta) shows the flipped-out position of the D2 domain for the p97ND1D2:ASPL complex. The lysine residue 315 and the bound SAH is shown as sticks with electron density around SAH. **f,** Refinement of the p97 residue Lys315 to verify the methylation state. The 2Fo-Fc map is contoured at 1σ (blue mesh) and the Fo-Fc difference map at 2.5σ (green mesh). **g,** Crystal packing of the p97:ASPL:METTL21D complex.

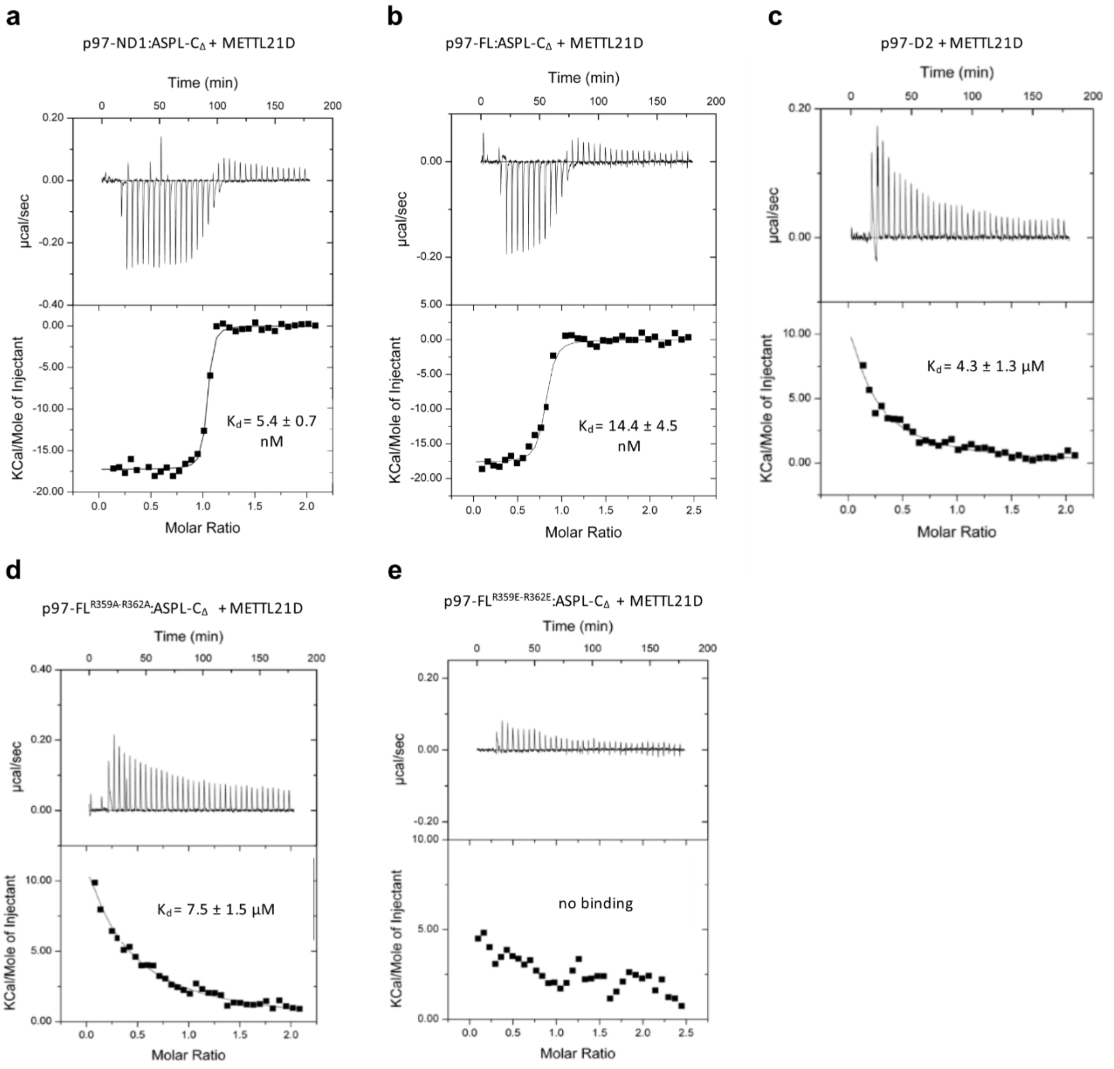

Supplementary Figure 2. ITC titration graphs. a,b, Titration of METTL21D to the p97-ND1:ASPL and p97:ASPL complexes reveals a comparable and high affinity. **c,** Although the p97-D2 domain contains an SRH-motif including two arginine residues, METTL21D binds to it only weakly. **d,** Mutation of the arginines present in the p97-D1 domain, Arg359 and Arg362, to alanines results in a thousand-fold weakening of the interaction between p97:ASPL and METTL21D. **e,** Mutation of p97 residues Arg359 and Arg362 to oppositely charged glutamates results in almost complete abrogation of the interaction between p97:ASPL and METTL21D. All results are summarized in the main text (Table 1).

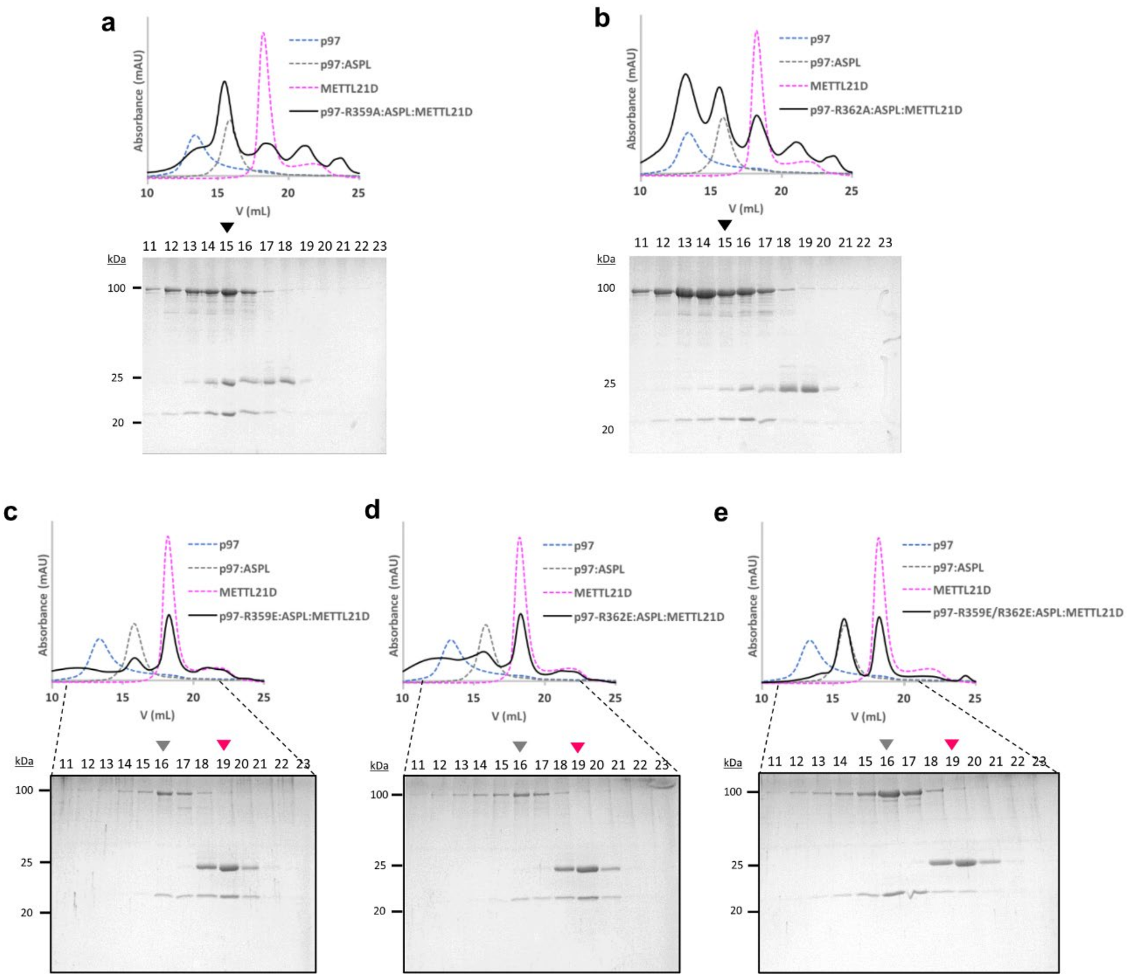

Supplementary Figure 3. Analytical SEC profiles of p97 interaction mutants. a, Mutation of the p97 residue Arg359 to alanine has a weak effect on binding of METTL21D, and the p97:ASPL:METTL21D complex can still form (▼). **b,** Mutation of the p97 residue Arg362 to alanine has a stronger effect on binding of METTL21D, so less of the complex can form, however there are still fractions containing p97, ASPL and METTL21D, indicating weaker interaction (▼). **c,** Mutation of Arg359 to an oppositely charged glutamate completely prevents p97:ASPL (▼) form interacting with METTL21D as indicated by the separate METTL21D and its occurrence in higher-volume fractions (▼). **d,** Mutation of Arg362 to an oppositely charged glutamate also completely prevents p97:ASPL (▼) from interacting with METTL21D (▼). **e,** Combining the single mutations to a double mutation of Arg359 and Arg362 to glutamates results in abrogating the interaction with METTL21D as well.

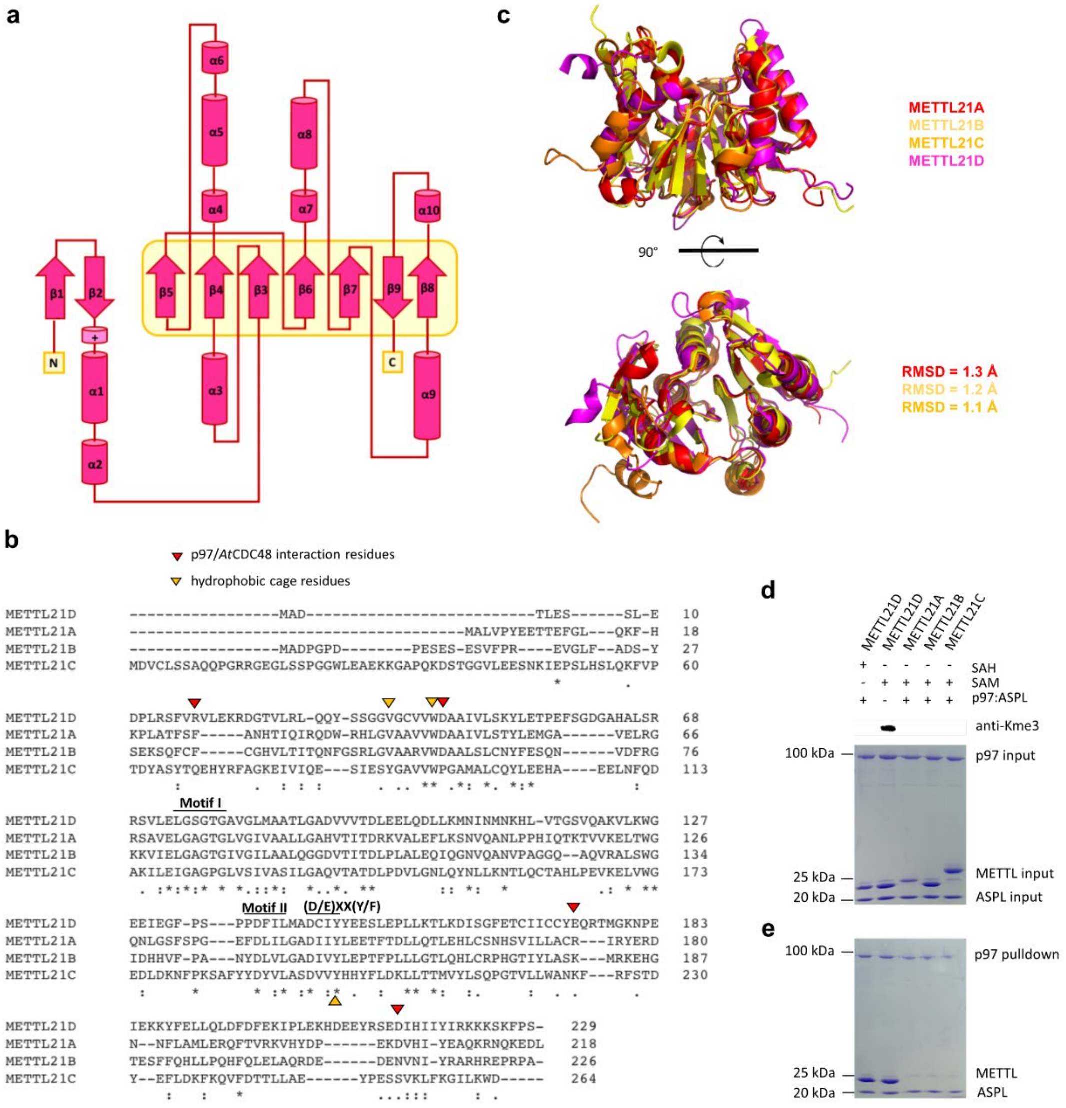

Supplementary Figure 4. Topology of METTL21D and structural similarity to related methyltransferases. a, 2D topology diagram of METTL21D shows the seven-β-strand protein core (yellow box) and the surrounding ten α helices and two additional β strands. The short α helix that appears upon METTL21D binding to p97 and closes the hydrophobic cage is marked with (+). **b,** Alignment of METTL21A-D shows no conservation of the p97-interacting residues (▼), which explains why related methyltransferases cannot trimethylate p97 despite the high structural similarity. On the contrary, the hydrophobic cage residues (▼) are highly conserved. **c,** Structural comparison reveals high structural similarity of METTL21D to METTL21A, METTL21B and METTL21C. **d,** Only METTL21D trimethylated p97 in presence of ASPL. **e,** Only METTL21D binds p97 in presence of ASPL (His-tagged p97, pulldown on Ni-NTA resin). ASPL binds in all instances, regardless of the added methyltransferase.

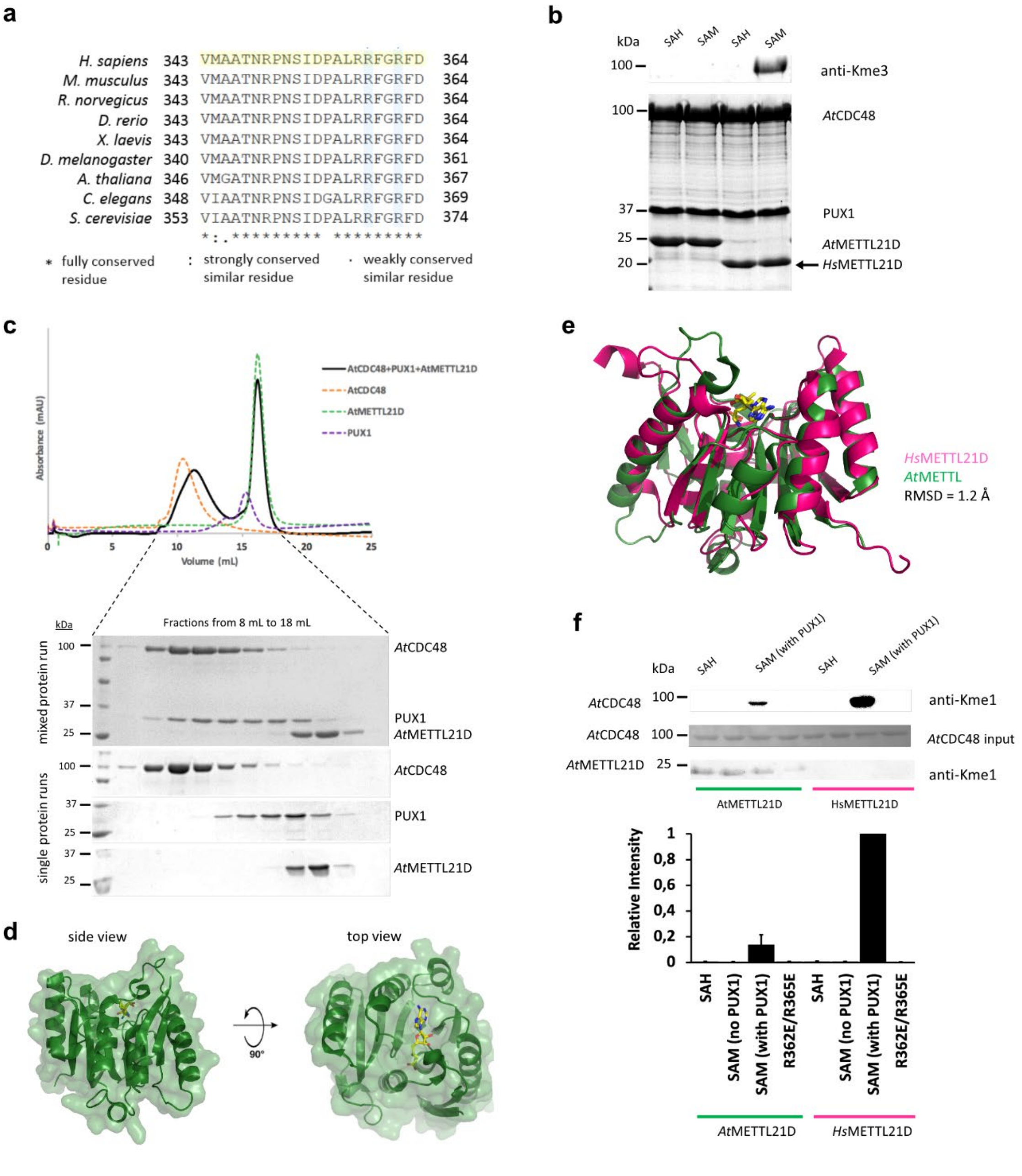

Supplementary Figure 5. Structure and activity of AtMETTL21D. a, Conservation of the SRH motif across species. **b,** *At*METTL21D cannot trimethylate *At*CDC48, whereas the human METTL21D can trimethylate *At*CDC48 cross-species. **c,** Analytical SEC shows weak binding of *At*METTL21D to *At*CDC48:PUX1. **d,** Crystal structure of the *Arabidopsis thaliana At*METTL21D with SAH bound. **e,** *At*METTL21D has a high degree of structural similarity to human METTL21D. **f,** *At*METTL21D can monomethylate *At*CDC48 in presence of PUX1, although the signal is weak compared to methylation by human METTL21D. The double-arginine mutant *At*CDC48$^{R362E/R365E}$ can abolish methylation by both methyltransferases.

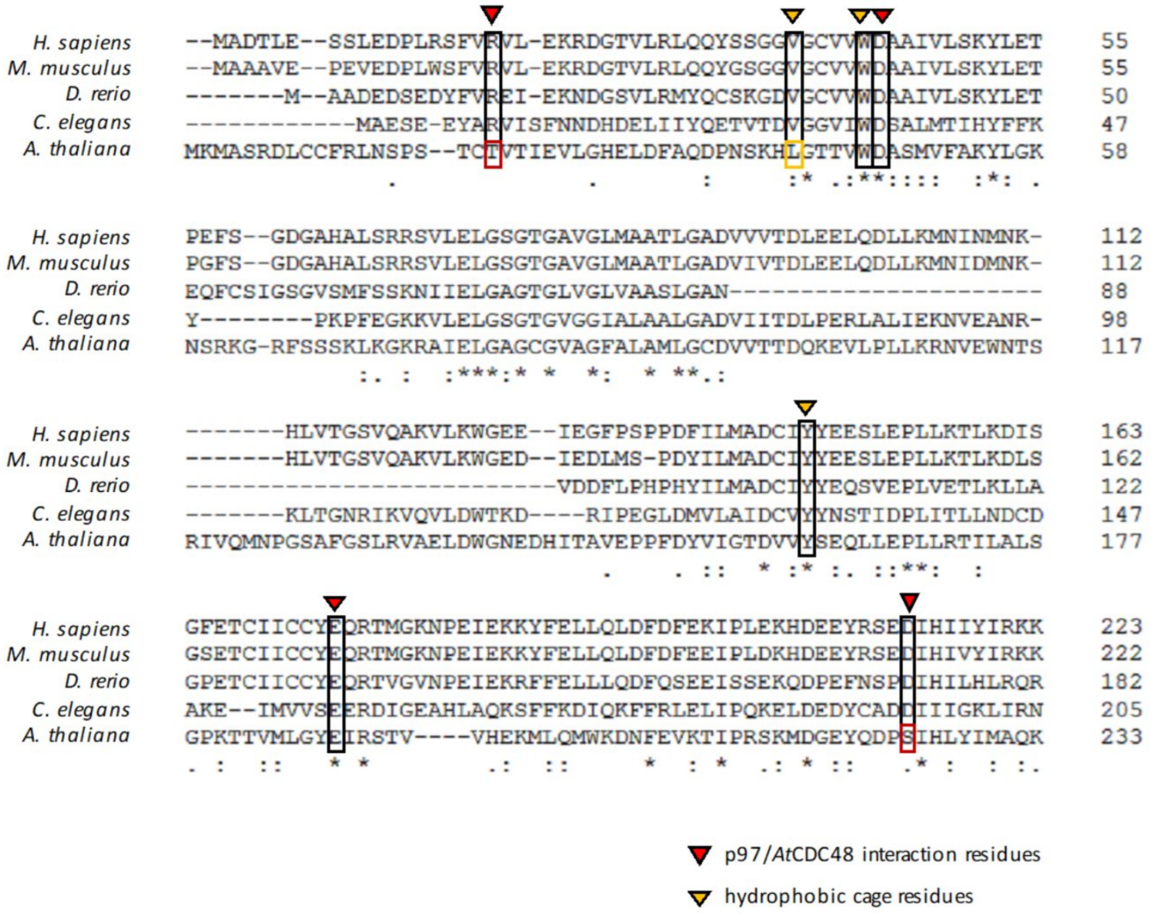

Supplementary Figure 6. Comparison of conserved p97 interaction residues across METTL21D orthologs. The newly discovered interaction residues (▼), as well as the hydrophobic cage residues (▼), are highly conserved in species ranging from human to *C. elegans*. However, the *A. thaliana* homolog of METTL21D shows a difference in two putative interaction residues (red boxes), as well as in the leucine residue that corresponds to the human Val38 in the hydrophobic cage (yellow box), which is likely to cause weaker binding to *At*CDC48 and reducing its capability to trimethylate its target.

Supplementary Movie S1. Movement of the METTL21D Val38 residue upon binding of METTL21D to p97. Upon binding of METTL21D (magenta) to p97 (blue) the METTL21D residue Val38 (represented as green spheres) moves approximately 8 Å towards the tri-methylated Lys315. This movement of Val38 results in closing of the hydrophobic cage around Lys315, together with residues Trp43 and Phe147 (upper and lower residues depicted as spheres in magenta, respectively). The crystal structure of the METTL21D monomer (PDB: 4LG1) was used as starting structure of unbound METTL21D.
